# Inhibitory Effect of Triterpenoids from *Dillenia serrata* (Dilleniaceae) on Prostaglandin E_2_ Production and Quantitative HPLC Analysis of Its Koetjapic Acid and Betulinic Acid Contents

**DOI:** 10.3390/molecules20023206

**Published:** 2015-02-16

**Authors:** Juriyati Jalil, Carla W. Sabandar, Norizan Ahmat, Jamia A. Jamal, Ibrahim Jantan, Nor-Ashila Aladdin, Kartiniwati Muhammad, Fhataheya Buang, Hazni Falina Mohamad, Idin Sahidin

**Affiliations:** 1 Drug Herbal Research Centre, Faculty of Pharmacy, Universiti Kebangsaan Malaysia, Jalan Raja Muda Abdul Aziz, Kuala Lumpur 50300, Malaysia; E-Mails: cw.sabandar@yahoo.com (C.W.S.); jamia@pharmacy.ukm.my (J.A.J.); ibj@pharmacy.ukm.my (I.J.); shila.aladdin@gmail.com (N.-A.A.); kartini_mohd@yahoo.com (K.M.); fhataheya_buang@yahoo.com (F.B.); hazni@pharmacy.ukm.my (H.F.M.); 2 Faculty of Applied Sciences, Universiti Teknologi Mara, Shah Alam 40450, Malaysia; E-Mail: noriz118@salam.uitm.edu.my; 3 Laboratory of Natural Products Chemistry, Faculty of Pharmacy, Halu Oleo University, Kendari 93232, Indonesia; E-Mail: sahidin02@yahoo.com

**Keywords:** Dilleniaceae, *Dillenia serrata*, triterpenoids, prostaglandin E_2_, HPLC

## Abstract

The crude methanol extracts and fractions of the root and stem barks of *Dillenia serrata* Thunb. showed 64% to 73% inhibition on the production of prostaglandin E_2_ (PGE_2_) in lipopolysaccharide-induced human whole blood using a radioimmunoassay technique. Three triterpenoids isolated from the root bark of the plant, koetjapic (**1**), 3-oxoolean-12-*en*-30-oic (**2**), and betulinic (**3**) acids, exhibited significant concentration-dependent inhibitory effects on PGE_2_ production with IC_50_ values of 1.05, 1.54, and 2.59 μM, respectively, as compared with the positive control, indomethacin (IC_50_ = 0.45 μM). Quantification of compounds **1** and **3** in the methanol extracts and fractions were carried out by using a validated reversed-phase high performance liquid chromatography (RP-HPLC) method. The ethyl acetate fraction of the stem bark showed the highest content of both compound **1** (15.1%) and compound **3** (52.8%). The strong inhibition of the extracts and fractions on cyclooxygenase-2 (COX-2) enzymatic activity was due to the presence of their major constituents, especially koetjapic and betulinic acids.

## 1. Introduction

Prostaglandin E_2_ (PGE_2_), a lipid mediator of prostanoids, is derived through the oxidative metabolism of arachidonic acid (AA) via the cyclooxygenase (COX) pathway [1]. PGE_2_ is abundantly produced in the human body and involved in controlling a variety of fundamental biological functions, including reproductive, neuronal, metabolic, and immune functions [1,2,3]. Despite of its constitutive functions, stimulation of PGE_2_ via the cyclooxygenase-2 (COX-2) pathway is recognized to be a pro-inflammatory mediator associated with inflammatory symptoms (*i.e.*, redness, swelling, and pain) [1,4]. These two opposing functions of PGE_2_ are mediated by the four E-prostanoid (EP) receptors, classified into the EP_1_ to EP_4_ subtypes [5,6]. Inhibition of PGE_2_ biosynthesis would therefore be expected to result in analgesic, anti-pyretic, and anti-inflammatory effects [7].

The genus *Dillenia* is comprised of about 100 species of shrubs and woody trees found in the seasonal tropics and subtropics of Asia, Australia, and Oceania [8]. Species of this family are used in traditional medicines [9], especially for gastrointestinal disorders [10]. The astringent preparation of the plants is used in the treatment of diarrhea [11]. Several species of *Dillenia* also produce edible fruits [8,12]. *Dillenia serrata* Thunb. is a small tree endemic to Indonesia (Sulawesi) [8,13] that produces a sweetish-acid edible fruit [8]. The vernacular names of *D. serrata* in Indonesia are Dongi (Manado), Dengilo (Manado), Dengen (Sulawesi), Songi (Sulawesi), and Menampa (Tembuku). *D. serrata* grows in the lowland primary forest in alluvial, sandy to clayey locations at 200 m above sea level [8,13]. Traditionally, the native make use of the fruit and stem bark of this plant, as well as the wood. The yellow fruit is used to acidify foods and can be consumed directly. Meanwhile, the decoction of stem bark is used orally to treat blood vomiting [14]. To the best of our knowledge, the phytochemistry and pharmacology of *D. serrata* have yet to be established and remain to be explored. Meanwhile, other *Dillenia* species such as *D. indica*, *D. papuana*, *D. pentagyna*, *D. philippinensis*, *D. parviflora*, and *D. retusa* have already been reported to contain several types of triterpenoids [15,16,17,18,19], flavonoids, and flavonoid glycosides [20,21,22], as well as phenolic compounds [21,23]. Bioactivities such as antimicrobial [18,24,25,26], anti-inflammatory [27], antinociceptive and antioxidant [26,28], antidiabetic and hypolipidemic [29], anti-leukemic [30], anti-tumor [23,31], anti-hypertension [32], and anti-protozoal [33] have been attributed to these species.

The present study is an attempt to isolate and elucidate the structure of the chemical constituents of *D. serrata*, as well as to examine their inhibitory activity on PGE_2_ production in human whole blood. In addition, we also quantified the detectable koetjapic acid (**1**) and betulinic acid (**3**) in the plant extracts, both of which have been reported to possess significant biological activities, including antibacterial [34,35], anticancer [30,36,37,38,39], anti-inflammatory [40,41] and anti-HIV properties [42,43].

## 2. Results and Discussion

### 2.1. Isolation and Characterization of Compounds

**Figure 1:**
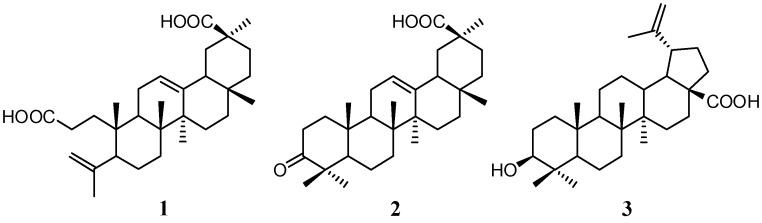
Chemical structure of the isolated triterpenoids from *D. serrata*.

After successive partition of the crude methanol extract of the root bark with petroleum ether and ethyl acetate, serial chromatography yielded two known oleanene-type triterpenoids, koetjapic acid (**1**) and 3-oxoolean-12-en-30-oic acid (**2**) and a lupene derivative, betulinic acid (**3**). Compound **3** was identified as a major compound of this plant. Koetjapic acid (**1**, also known as a *seco*-triterpenoid) was unexpectedly identified in the extracts of *D. serrata*. This compound was first reported from *Sandoricum koetjape* (Meliaceae) by Kaneda *et al.* [44] and, to the best of our knowledge, the occurrence of koetjapic acid (**1**) in *Dillenia* species has not been previously reported. The presence of *seco*-triterpenoids seems to be a common feature of *Dillenia* species, as reported by previous works [18,19]. Figure 1 displays the structures of these isolated triterpenoids. Identification of all triterpenoids was accomplished using a combination of physicochemical and spectroscopic experiments, *viz*. IR, 1D NMR as well as 2D NMR and HRESI-MS. All obtained values were similar to those reported in the literature [18,44,45].

### 2.2. Quantitative Analysis of Koetjapic Acid (**1**) and Betulinic Acid (**3**)

Previous HPLC studies on quantification of betulinic acid (**3**) in plants have been reported in some papers [46,47,48]. Bae *et al.* [46] quantified betulinic acid (**3**) in *Ziziphus fructus* using a Nova-Pak C_18_ column, eluted with phosphate buffer (Na_2_HPO_4_ 0.05 M, pH 2.5)-methanol at a ratio of 19:81 and showed retention time at 28 min. Considering that the background noise resulted from methanol, Oliveira *et al.* [47] improved the quantification of this compound in *Doliocarpus schottianus* by using acetonitrile instead of methanol. The condition was isocratic with acetonitrile-water pH 3.0 (9:1) and the retention time was at 11.5 min. Kumar *et al.* [30] applied this method for quantification of betulinic acid (**3**) from *D. indica* in a study on anticancer activity of this plant. A modification of the method was also performed using a Diamonsil C_18_ column, eluted with acetonitrile-water (86:14) for quantification of betulinic acid (**3**) in *Betula platyphylla* and showed that the retention time was at 16.5 min [48]. To the best of our knowledge, quantitative analysis of koetjapic acid (**1**) has not previously been reported.

**Figure 2:**
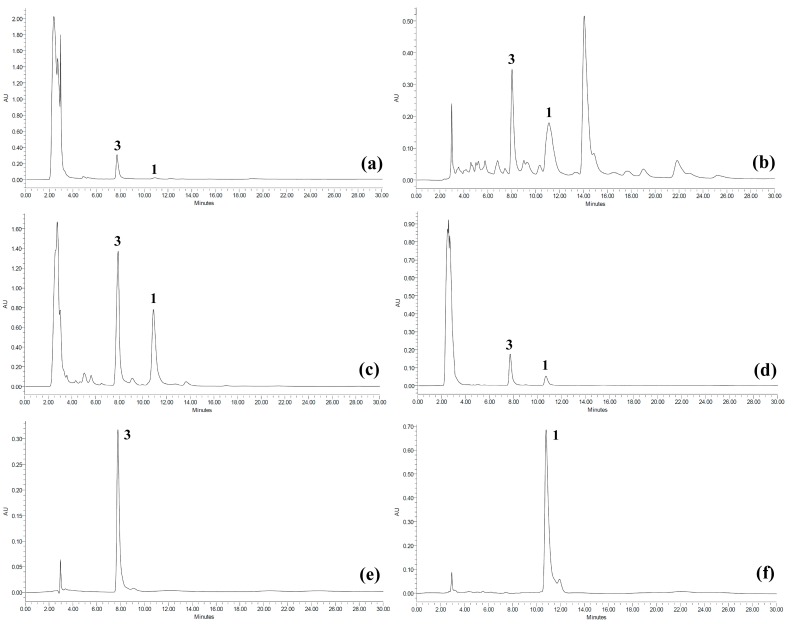
HPLC chromatograms for (**a**) crude MeOH extract, (**b**) petroleum ether fraction, (**c**) ethyl acetate fraction, (**d**) MeOH fraction, (**e**) isolated betulinic acid (**3**) and (**f**) isolated koetjapic acid (**1**).

In our study, a pH modification of a method [47] was conducted in order to shorten the retention time of betulinic acid (**3**), resulting in a rapid analysis. The method was also able to give a good separation for koetjapic acid (**1**) at a retention time of 10.801 min (see Figure 2). Validation of the reversed phase HPLC method for quantification of these compounds was determined by regression equation, coefficient correlation (*r*^2^), limit of detection (LOD) and limit of quantification (LOQ) (see Experimental Section). The calibration curves plotted were linear over the concentration range of 62.5 to 1000 μg/mL. LOD and LOQ values were found to be reliable for the method according to Bretnall *et al.* [49]. In addition, the precision of HPLC method regarding reproducibility and repeatability was satistacfory as indicated by the relative standard deviation (RSD) not greater than 5.0% [50] of peak area and retention time by intraday and interday analyses. Thus, the linearity, precision and accuracy of this modification were acceptable for quantitative analysis of koetjapic acid (**1**) and betulinic acid (**3**) in *D. serrata*.

Distribution of detectable amounts of koetjapic acid (**1**) and betulinic acid (**3**) in the crude methanol extracts and fractions of *D. serrata* was determined by using the validated HPLC method. As shown in Table 1, koetjapic acid (**1**) and betulinic acid (**3**) were mainly concentrated in the ethyl acetate fraction while they were found in least amounts in the methanol fractions. From the quantitative analysis, we also found that betulinic acid (**3**) preferentially accumulated in the stem bark rather than the root bark. Koetjapic acid (**1**) together with betulinic acid (**3**) are suggested as the chemical markers of *D. serrata*. Betulinic acid has been reported from several genera of the Dilleniaceae family, including *Dillenia*, *Wormia*, and *Acrotrema* [16]. The presence of a considerable amount of betulinic acid in these genera suggests that this compound may be a chemotaxonomic marker of the family. Koetjapic acid (**1**) can also be used as an additional marker since its presence in this plant seems unique among the *Dillenia* species.

**Table 1:** Concentrations of koetjapic acid (**1**) and betulinic acid (**3**) in the crude methanol extracts and fractions of *Dillenia serrata*.

Sample	Code	Concentration of 1 (mg/g) *	Concentration of 3 (mg/g) *
*Root bark*			
Crude MeOH	CRB	21.31 ± 1.99	81.76 ± 1.40
Petroleum ether fraction	RB-PEF	46.32 ± 0.61	128.20 ± 3.91
Ethyl acetate fraction	RB-EAF	117.62 ± 1.54	424.26 ± 2.97
MeOH fraction	RB-MF	19.78 ± 1.41	90.93 ± 2.53
*Stem bark*			
Crude MeOH	CSB	4.27 ± 1.09	102.59 ± 2.99
Petroleum ether fraction	SB-PEF	45.37 ± 1.68	137.58 ± 3.09
Ethyl acetate fraction	SB-EAF	151.29 ± 2.40	528.08 ± 1.11
MeOH fraction	SB-MF	8.49 ± 0.50	58.81 ± 1.21

* Value given in mean ± SD (*n* = 3).

### 2.3. Inhibition of Prostaglandin E_2_ (PGE_2_)

Production of PGE_2_ induced by lipopolysaccharide (LPS) in human whole blood has been measured as a reflection of cyclooxygenase-2 (COX-2) activity of blood cells such as monocytes [51]. The inhibition of PGE_2_ production in human whole blood can be expressed as an inhibition of the enzymatic activity of COX-2 and/or inhibition of the expression of COX-2 protein. Crude methanol extracts and fractions of *D. serrata* root and stem bark were investigated for their ability to inhibit PGE_2_ in LPS-induced human whole blood and showed various percentage inhibitions at a concentration of 10 μg/mL (Table 2). This inhibitory activity indicated the presence of bioactive compounds in the crude methanol extracts and fractions of *D. serrata*.

**Figure 3:**
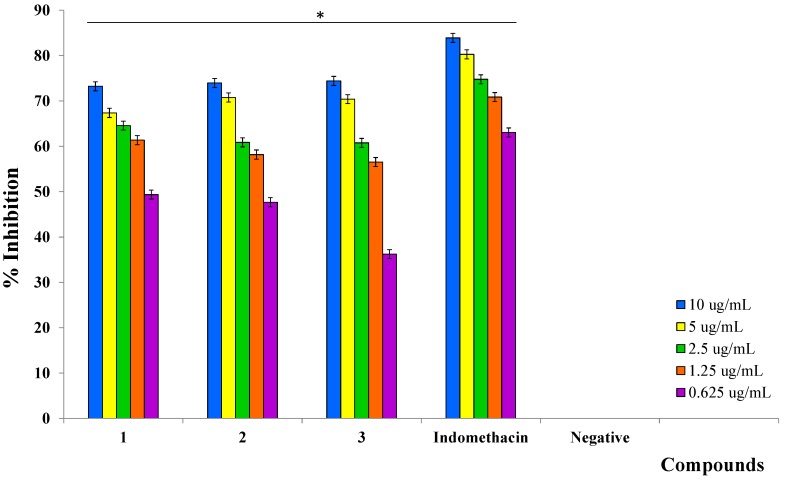
Inhibitory effects of compounds **1**, **2**, and **3** on PGE_2_ production by LPS-stimulated human whole blood at various concentrations (value given in mean ± SD; *n* = 3; * *p* < 0.05).

The three triterpenoids **1**–**3** isolated from the root bark of *D. serrata* were also able to inhibit PGE_2_ production induced by LPS. This concentration-dependent inhibition was observed at five serial concentrations ranging from 10 to 0.625 μg/mL (Figure 3). All compounds showed significant inhibitory activity on PGE_2_ production as indicated by their IC_50_ values (Table 3). Of the three compounds, koetjapic acid (**1**) was identified as a promising inhibitor, with an IC_50_ value of 1.05 μM, comparable to that of a potent cyclooxygenase inhibitor, indomethacin (0.45 μM). In contrast to our study, koetjapic acid (**1**) was considered inactive in mouse ear inflammation model induced by tetradecanoylphorbol acetate [52]. Betulinic acid (**3**) had a higher IC_50_ value (2.59 μM) among the three compounds. The inhibition of LPS-induced PGE_2_ production by betulinic acid has been reported in human peripheral blood mononuclear cells (hPBMCs), and this inhibition was due to the suppression of COX-2 protein expression induced by LPS [53]. On the other hand, Wenzig *et al.* [54] investigated inhibition of COX-2 enzyme activity by betulinic acid and the results showed that the inhibition against this enzyme was not significant (IC_50_ ˃ 125 μM). Based on both studies, we can suggest that betulinic acid (**3**) plays role as an inhibitor of LPS induced expression of COX-2 protein, hence it can inhibits the production of PGE_2_ induced by LPS. Koetjapic acid (**1**) and 3-oxoolean-12-en-30-oic acid (**2**) may also probably inhibiting the COX-2 enzyme by similar mechanisms. However, phospholipase A_2_ (PLA_2_) may also be a potential target for these three compounds.

**Table 2:** Percentage inhibition (%) and IC_50_ values of the crude MeOH extracts and fractions of *D. serrata* on production of PGE_2_ in LPS-induced human whole blood.

Sample	Code	% Inhibition (10 µg/mL) *^,†^	IC_50_ (µg/mL) *
*Root bark*			
Crude MeOH	CRB	73.03 ± 0.77	1.80 ± 0.09
Petroleum ether fraction	RB-PEF	72.36 ± 1.18	0.23 ± 0.15
Ethyl acetate fraction	RB-EAF	73.86 ± 2.90	2.00 ± 0.13
MeOH fraction	RB-MF	65.01 ± 0.21	3.33 ± 0.53
*Stem bark*			
Crude MeOH	CSB	71.88 ± 1.80	0.96 ± 0.32
Petroleum ether fraction	SB-PEF	64.26 ± 0.98	4.61 ± 0.09
Ethyl acetate fraction	SB-EAF	64.06 ± 1.62	1.31 ± 0.33
MeOH fraction	SB-MF	69.02 ± 0.90	1.24 ± 0.25
Indomethacin (positive control)		83.90 ± 0.27	0.16 ± 0.02

* Value given in mean ± SD (*n* = 3); **^†^** *p* < 0.05.

Since betulinic acid (**3**) was found as a major compound in all the crude extracts and fractions, the LPS-induced PGE_2_ production inhibitory activity might be contributed mainly by this compound. However, as shown in Table 2, various IC_50_ values of the crude methanol extracts and fractions of *D. serrata* were found not to correlate with either the amounts of koetjapic acid (**1**) or betulinic acid (**3**). For example, both RB-PEF and SB-PEF fractions contained almost the same concentrations of compound **1** and **3**, but gave very different IC_50_ values (0.23 and 4.61 μg/mL, respectively) on inhibition of LPS-induced PGE_2_ production. This result indicated that there may be other potent inhibitors present in fraction RB-PEF. Similarly, fractions SB-EAF and SB-MF possessed close IC_50_ values (1.31 and 1.24 μg/mL, respectively), but they contained very different amounts of compounds **1** and **3**. Interestingly, fraction RB-MF with IC_50_ of 3.33 μg/mL contains about 0.06 μg (1.98%) of compound **1** and 0.30 μg (9.09%) of compound **3**, and these amount were considerably lower than the IC_50_ values of pure compound **1** and **3**. Based on these results, we suggest that substantial amounts of koetjapic (**1**) and betulinic (**3**) acids in the crude methanol extracts and fractions of *D. serrata* may affect LPS-induced PGE_2_ production. However, the presence of other compounds in the extracts and fractions may also contribute to the activity, either in inhibiting or assisting LPS-induced PGE_2_ production.

**Table 3:** IC_50_ values of compounds **1**, **2**, and **3** on PGE_2_ production by LPS-stimulated human whole blood.

Compounds	IC_50_ (µM) *
**1**	1.05 ± 0.02
**2**	1.54 ± 0.05
**3**	2.59 ± 0.07
Indomethacin (positive control)	0.45 ± 0.02

* Value given in mean ± SD (*n* = 3).

Koetjapic acid (**1**) has various pharmacological activities, including growth inhibition of multi-drug resistant bacteria *Staphylococcus aureus* and *Pseudomonas aeruginosa* (minimum inhibitory concentration, MIC of 12.5 and 6.25 μg/mL, respectively) [34], inhibition on Epstein-Barr virus antigen activation [36] and inhibition of DNA polymerase beta (IC_50_ 20 μM) [37]. This compound also preserved high viability of the Raji cells [36]. Meanwhile, Nick *et al.* [18] investigated the antibacterial activity of 3-oxoolean-12-*en*-30-oic acid (**2**) from *D. papuana* that inhibited the growth of *Bacillus subtilis*, *Escherichia coli*, and *Micrococcus luteus*. Some papers have reported the anti-inflammatory, antileukemic and anti-HIV activities of betulinic acid (**3**) [38,39,40,41,42,43]. Treatment of hPBMCs with this compound alone did not elicit any PGE_2_ production and was non-cytotoxic, as indicated by trypan blue assay [53].

## 3. Experimental Section

### 3.1. General Information

Standard PGE_2_, Anti-PGE_2_ sera (developed in rabbit), lipopolysaccharide (LPS) from *E. coli* 0111:B4 and dextran from *Leuconostoc mesentroides* were purchased from Sigma (St. Louis, MO, USA). Radiolabelled PGE_2_ ([^3^H]-PGE_2_; 50 μCi/mmol) and scintillation cocktail (Ultima Gold MV) were purchased from PerkinElmer (Boston, MA, USA). Bovine serum albumin (BSA), ethylenediamine tetraacetic acid (EDTA), activated charcoal and analytical and HPLC grades of solvents (petroleum ether, ethyl acetate, methanol, and acetonitrile) were purchased from Merck (Darmstadt, Germany). Chromatography techniques (Thin Layer Chromatography; TLC, Vacuum Liquid Chromatography; VLC and Column Chromatography; CC) were performed using Merck Si-gel. Melting point was observed using a Stuart Melting Point SMP10. UV-Vis and IR spectra were recorded on a Shimadzu UV1800 and a PerkinElmer GX IR (ATR), respectively. High-resolution electrospray ionization mass spectrometry (HRESI-MS) was performed on a Bruker MicroTOF-Q mass spectrometer. Spectra of ^1^H-NMR (600 MHz) and ^13^C-NMR (150 MHz) were recorded on a Bruker Advance NMR. Radioactivity was measured using a Tri-Carb 3110 TR PerkinElmer Liquid Scintillation Analyzer. HPLC technique was performed on a Waters 2535 Quaternary Gradient Module HPLC using an XBridge^TM^ RP C-18 column (4.6 × 250 mm, 5 μm).

### 3.2. Plant Material

The barks of root and stem of *D. serrata* were collected from secondary forest in Onewila village, a region of Southeast Sulawesi, Indonesia and authenticated by Herbarium Bogoriense, Bogor, Indonesia (voucher number: BO-1902181).

### 3.3. Extraction and Isolation of Compounds

The dried root barks (1.15 kg) and stem barks (2.5 kg) were macerated with MeOH (4 and 8 L, respectively) for 24 h. The extracts were filtered and the solvent was evaporated under vacuum. The steps were executed three times to yield 500 g crude (43.5%) extract of root barks (CRB) and 600 g crude (24%) extract of stem barks (CSB). 250 g of CRB was dissolved in methanol (2.5 L) and left to stand overnight to re-crystallize compound **3**. Compound **3** (993.4 mg) was collected using a vacuum filter and the residue was evaporated. The remaining CRB was dissolved in a small volume of MeOH (~100 mL) and partitioned three times with petroleum ether (PE) and ethyl acetate (EtOAc) (~500 mL), to give PE (1.5 g) and EtOAc (29.7 g) soluble fractions, respectively. The PE fraction was subjected onto VLC with hexane-EtOAc to give 28 sub-fractions. Sub-fractions were combined into 7 fractions (F_1_–F_7_) based on TLC analysis. Compound **2** (9.0 mg) was precipitated from F_3_. The EtOAc fraction was then subjected onto VLC with hexane-EtOAc to yield 32 sub-fractions. Compound **1** (102.2 mg) was precipitated from sub-F_29_ and sub-F_31_. For HPLC and bioactivity samples, 10 g of each crude extract was suspended in methanol and partitioned successively with petroleum ether, ethyl acetate and methanol. Six fractions were then evaporated under vacuum to yield residues of petroleum ether (RB-PEF) (0.6 g, 6%), ethyl acetate (RB-EAF) 1.9 g, 19%), and methanol (RB-MF) (7.2 g, 72%) fractions of root barks followed by petroleum ether (SB-PEF) (0.3 g, 3%), ethyl acetate (SB-EAF) (1.7 g, 17%), and methanol (SB-MF) (7.9 g, 79%) fractions of stem barks. These residues were stored in a refrigerator at 4 °C until analyses.

*Koetjapic acid (3,4-seco-olean-4(23), 12-diene-3,30-dioic acid)* (**1**); 102.2 mg; white prisms (MeOH); mp 296–298°; UV (EtOH) λ_max_ nm (log ε): 203 (3.88); IR (ATR) ʋ_max_ cm^−1^: 3440, 2978, 2860, 1706, 1702, 1698, 1694, 1454, 1387, 1281, 1230, 1192, 906; HRESI-MS *m/z*: [M+Na]^+^ 493.3279 (calc. for C_30_H_46_O_4_, 470.3396). ^1^H-NMR (600 MHz; DMSO-*d*_6_) δ_H_ (ppm): 0.74 (*s*, H_3_-28), 0.84 (H_α_-16), 0.88 (*s*, H_3_-25), 0.96 (*s*, H_3_-26), 0.99 (*bs*, H_α_-15), 1.07 (*s*, H_3_-29), 1.15 (*s*, H_3_-27), 1.25 (H_α_-7), 1.27 (H_2_-22), 1.31 (H_2_-6), 1.39 (H_α_-2), 1.45 (H_β_-2), 1.52 (H_β_-7), 1.61 (H_α_-19), 1.70 (H_β_-15), 1.72 (*s*, H_3_-24), 1.73 (H_β_-19), 1.76 (H_α_-9), 1.78 (H_α_-11, H_2_-21), 1.89 (H_β_-11, H_β_-18), 1.99 (H_α_-5, H_β_-16), 2.06 (*m*, H_α_-1), 2.26 (*m*, H_β_-1), 4.66 (*s*, H_a_-23), 4.85 (*s*, H_b_-23), 5.18 (*t*, H-12) and 12.04 (2OH-3,30); ^13^C-NMR (150 MHz; DMSO-*d*_6_) δ_C_ (ppm): 17.0 (C-26), 19.6 (C-25), 23.6 (C-11), 23.9 (C-24), 24.5 (C-6), 26.0 (C-27), 26.2 (C-15), 26.8 (C-16), 28.5 (C-1), 28.6 (C-28), 28.7 (C-29), 31.1 (C-21), 31.3 (C-7), 32.1 (C-17), 34.3 (C-2), 37.7 (C-9), 38.5 (C-22), 39.1 (C-10), 39.5 (C-8), 42.1 (C-14), 42.8 (C-19), 43.6 (C-20), 48.3 (C-18), 49.8 (C-5), 113.9 (C-23), 122.2 (C-12), 144.8 (C-13), 147.6 (C-4), 175.3 (C-3) and 178.5 (C-30). NMR spectral data were identical to those given in [44].

*3-Oxoolean-12-en-30-oic acid* (**2**); 9 mg; white crystalline solid (MeOH); mp 270–272°; UV (EtOH) λ_max_ nm (log ε): 205 (4.00); IR (ATR) ʋ_max_, cm^−1^: 3498; 2968–2861; 1705; 1698; 1695; HRESI-MS *m/z*: [M+Na+16]^+^ 493.3300 (calc. for C_30_H_46_O_3_, 454.34470); ^1^H-NMR (600 MHz; DMSO-*d*_6_) δ_H_ (ppm): 0.74 (*s*, H_3_-28), 0.86 (H_α_-16), 0.96 (*s*, H_3_-24, H_3_-26), 0.99 (*s*, H_3_-25), 0.99 (H_α_-15), 1.00 (*s*, H_3_-23), 1.06 (*s*, H_3_-29), 1.13 (*s*, H_3_-27), 1.29 (H_α_-21, H_2_-22), 1.33 (H_α_-5), 1.35 (H_α_-7), 1.41 (H_α_-1), 1.46 (H_2_-6), 1.53 (H_β_-7), 1.60 (H_α_-19), 1.64 (H_α_-9), 1.70 (H_β_-19), 1.73 (H_β_-15), 1.79 (H_β_-21), 1.80 (H_β_-1), 1.88 (H_2_-23), 1.91 (H_β_-18), 1.98 (H_β_-16), 2.29 (H_α_-2), 2.53 (H_β_-2) and 5.19 (*t*, H-12) and 12.10 (*bs*, OH-30); ^13^C-NMR (150 MHz; DMSO-*d*_6_) δ_C_ (ppm): 15.3 (C-25), 16.9 (C-26), 19.6 (C-6), 21.6 (C-24), 23.6 (C-11), 26.0 (C-27), 26.2 (C-15), 26.7 (C-23), 26.8 (C-16), 28.6 (C-28), 28.7 (C-29), 31.1 (C-21), 32.0 (C-17), 32.1 (C-7), 34.2 (C-2), 36.6 (C-10), 38.5 (C-22), 39.0 (C-1), 39.7 (C-8), 41.7 (C-14), 42.8 (C-19), 43.6 (C-20), 46.6 (C-9), 47.2 (C-4), 48.3 (C-18), 54.7 (C-5), 122.2 (C-12), 144.9 (C-13), 178.5 (C-30) and 216.7 (C-3). NMR spectral data were identical to those in [18].

*Betulinic acid (3β-hydroxy-20(29)-lupen-28-oic acid)* (**3**); 993.4 mg; white crystalline needles (MeOH); mp 296–301°; UV (EtOH) λ_max_ nm (log ε): 206 (3.96); IR (ATR) ʋ_max_, cm^−1^: 3446, 2940; 2870, 1684, 1681, 1456, 1360, 1236, 1043, 886; HRESI-MS *m/z*: [M−H]^−^ 455.35252 (calc. for C_30_H_48_O_3_, 456.36035.); ^1^H-NMR (600 MHz; DMSO-*d*_6_) δ_H_ (ppm): 0.64 (*s*, H_3_-24), 0.75 (*s*, H_3_-26), 0.84 (H_α_-1), 0.85 (*s*, H_3_-25), 0.86 (*s*, H_3_-23), 0.92 (*s*, H_3_-27), 0.97 (H_α_-12), 1.09 (H_α_-15), 1.15 (H_β_-11), 1.24 (H_α_-9), 1.30 (H_β_-6, H_β_-7, H_α_-21), 1.37 (H_α_-11, H_α_-16), 1.44 (H_2_-2, H_α_-6, H_β_-15, H_α_-22), 1.50 (*t*, H_α_-18), 1.55 (H_β_-1), 1.60 (H_β_-12), 1.64 (*s*, H_3_-30), 1.79 (H_β_-21, H_β_-22), 2.11 (H_β_-16), 2.21 (*td*, *J*_1_ = *J*_3_ = 3.6, *J*_2_ = 2.4, H_β_-13), 2.95 (H_α_-3, H_β_-19), 4.30 (*bs*, OH-3), 4.55 (*d*, *J* = 0.6, H_a_-29), 4.68 (*d*, *J* = 1.8, H_b_-29) and 12.10 (*bs*, OH-28); ^13^C-NMR (150 MHz; DMSO-*d*_6_) δ_C_ (ppm): 14.8 (C-27), 16.2 (C-25), 16.3 (C-24), 16.4 (C-26), 18.4 (C-6), 19.4 (C-30), 20.9 (C-11), 25.5 (C-12), 27.6 (C-2), 28.6 (C-23), 29.7 (C-15), 30.5 (C-21), 32.2 (C-16), 34.4 (C-7), 36.8 (C-22), 37.2 (C-10), 38.0 (C-13), 38.7 (C-1), 38.9 (C-4), 40.7 (C-8), 42.5 (C14), 47.1 (C-19), 48.9 (C-18), 50.4 (C-9), 55.3 (C-5), 55.9 (C-17), 77.2 (C-3), 110.1 (C-29), 150.8 (C-20) and 177.7 (C-28). NMR spectral data were identical with reference [45].

### 3.4. Quantification of Koetjapic Acid (**1**) and Betulinic Acid (**3**) Using HPLC

HPLC analysis was performed based on the method described by Oliveira *et al.* [47] with slight modification. HPLC (Waters 2535) equipped with a reversed-phased column C-18 (4.6 × 250 mm, 5 μm; XBridge, Waters, Dublin, Ireland) and photodiode array detector (Waters 2998) were used. Koetjapic acid (**1**) and betulinic acid (**3**) (1 mg/mL each) in methanol were injected (20 μL) three times and separated isocratically with acetonitrile-water (9:1) pH 2.5 (with trifluoroacetic acid) at a flow rate of 1 mL/min and 3000–3500 psi pressure. The compounds were detected at 210 nm [30,49].

The HPLC method for koetjapic acid (**1**) and betulinic acid (**3**) was validated by determination of linearity, precision and accuracy in accordance with ICH guidelines [55]. Linearity was evaluated from the linear regression equation and correlation coefficient (*r*^2^) of calibration curves constructed for both compounds within the concentration range of 62.5 to 1000 μg/mL (Table 4).

**Table 4:** Validation parameters of HPLC method for koetjapic acid (**1**) and betulinic acid (**3**).

Compound	Conc. ^1^	Intra-day precision ^2^						Inter-day precision ^4^		Equation (*r*^2^)	LOD ^5^	LOQ ^5^
		Rt ^3^			Area							
		Day 1	Day 2	Day 3	Day 1	Day 2	Day 3	Rt ^3^	Area			
Koetjapic acid (**1**)	62.5	1.51	2.16	0.06	4.51	3.62	3.68	1.20	1.48	*y* = 13523*x* − 44326 (0.9994)	1.89	5.75
	125	2.58	2.52	1.48	3.09	1.15	2.08	3.57	1.89			
	250	2.50	0.56	1.57	1.33	0.95	4.04	2.26	3.41			
	500	0.68	1.98	0.56	1.58	2.04	2.19	3.82	2.11			
	1000	0.94	0.83	1.74	1.62	1.27	1.59	2.49	2.66			
Betulinic acid (**1**)	62.5	0.48	0.44	1.31	4.92	3.82	3.45	2.29	2.46	*y* = 4851*x* − 42307 (0.9999)	9.23	27.97
	125	1.07	1.50	1.11	4.53	3.98	2.28	2.18	3.53			
	250	0.50	0.88	0.66	1.03	2.83	2.82	2.40	3.32			
	500	1.69	1.07	1.57	1.15	2.08	4.96	2.55	2.58			
	1000	0.83	0.89	0.83	2.86	2.57	2.86	1.55	4.03			

^1^ μg/mL; ^2^ *n* = 3; ^3^ Retention time; ^4^ *n* = 9; ^5^ ng/mL.

Precision was determined by the LOD and LOQ by injecting a series of known concentrations of the compounds. The values of LOD and LOQ were calculated from the relative standard deviation (RSD) and slope (*S*) of the calibration curves. The accuracy of the method regarding reproducibility and repeatability was evaluated by intra- and inter-day variation on three consecutive days with three repetitions each. The reproducibility and repeatability were demonstrated by the RSD of peak area and retention time.

The content of koetjapic acid (**1**) and betulinic acid (**3**) in the *D. serrata* extracts and fractions was quantified using the validated HPLC method. Precise amount of samples (10 mg for each of CRB, RB-PEF, RB-EAF, RB-MF, CSB, SB-PEF, SB-EAF, and SB-MF) were sonicated in methanol (1 mL) and filtered through a 0.45 μm filter. An aliquot of 20 μL of each sample was injected onto the HPLC.

### 3.5. Radioimmunoassay for Prostaglandin E_2_ (PGE_2_)

The inhibition of PGE_2_ production indicated by the concentration of PGE_2_ in human whole blood was measured according to the validated radioimmunoassay (RIA) method [51]. The application of human blood was permitted by the Ethics Committee of Universiti Kebangsaan Malaysia (UKM) with approval number NF-016-2013.

Human whole blood was drawn using aseptic vein puncture from the same donors of healthy volunteers when they had not taken any medicine or supplements during the last two weeks and fasted for 8 h prior to blood being withdrawn. The blood sample was prevented from coagulation by adding 10% (*v*/*v*) of 2% EDTA in a polypropylene tube. Duplicate 1 mL aliquots of EDTA-whole blood samples were transferred into test tubes and incubated with 10 μL of sample or indomethacin (1 mg/mL in 1:1 of DMSO-ethanol) for 15 min (37 °C) before LPS addition. The effects of samples or indomethacin on PGE_2_ production were studied by incubating each sample with whole blood-EDTA in the presence of LPS (10 μg/mL in 0.9% normal saline) for 24 h. For IC_50_, the concentration of samples were adjusted in five serial dilutions over a concentration range of 0.625 to 10 μg/mL. After incubation at 37 °C for 24 h, the plasma was separated by centrifugation at 2600 × g for 15 min at 4 °C. RIA buffer (phosphate buffered saline [0.01 M, pH 7.4] containing 0.1% BSA and 0.1% sodium azide) was used as the standard diluent of the assay. The plasma (100 μL) was added to anti-PGE_2_ (100 μL; diluted with ratio of 1:50,000) and [^3^H]-PGE_2_ (100 μL; 5000 cpm) and incubated for 18–24 h at 4 °C. After incubation, dextran-charcoal (200 μL) was added to the mixture and incubated for 10 min at 0 °C. The supernatant was then separated by centrifugation at 3000× *g* for 15 min at 4 °C and pipetted (300 μL) into liquid scintillation cocktail (3 mL). The radioactivity was measured using a liquid scintillation analyzer.

Concentration of PGE_2_ (pg/0.1 mL) in the blood was calculated using a semi-logarithmic graph of standard PGE_2_. Previously, standard PGE_2_ (1 mg/mL) had been serially diluted to concentrations ranging from 2.45 to 400 pg/0.1 mL. The interference of compounds in the crude methanol extracts and fractions towards RIA method was checked by adding crude extracts and fractions to the standards and found not to interfere with the measurements. The average count per min (cpm) values of standards and samples (B) resulting from antibody-antigen (labeled PGE_2_) binding in the plasma were subtracted from the non-specific binding (N_c_) together with the total binding between antibody and antigen (B_o_). The normalized percent bound (% B/B_o_) was then determined using Equation (1):

(1) $$\%\frac{B}{B_{o}}=\left(\frac{B-N_{c}}{B_{O}-N_{c}}\right)100$$

(2) $$\text{\% I}=\left(1-\frac{\left[{\text{Concentration of PGE}}_{2}\text{ in samples or standard}\right]}{\left[{\text{Concentration of PGE}}_{2}\text{ in control}\right]}\right)100\%$$

The calculated % B/B_o_ values were plotted against their respective concentrations of standard PGE_2_ in picograms (pg) semi-logarithmically. Thus, the interpolation of % B/B_o_ values for samples and indomethacin using PGE_2_ standard curve resulted in the determination of PGE_2_ concentration in the blood. The percentage inhibition (% I) was then calculated using Equation (2).

### 3.6. Statistical Analysis

The HPLC analysis and bioassay were performed in triplicate and the data were expressed as means ± SD. Empower software (Waters) was used to construct a calibration curve of koetjapic and betulinic acids as well as their quantification. The bioactivity data were analyzed using Statistical Package for Social Sciences (SPSS) software Version 17. Data were analyzed using one way ANOVA analysis with a probability *p* < 0.05 representing a significant difference as compared to control. GraphPad Prism 5 was used to determine the IC_50_ value of active extracts and compounds.

## 4. Conclusions

Our study revealed that *D. serrata* possesses a promising inhibitory effect on LPS-induced PGE_2_ production. Three triterpenoids from this plant, koetjapic acid (**1**), 3-oxoolean-12-en-30-oic acid (**2**), and betulinic acid (**3**), were found to inhibit LPS-induced PGE_2_ production concentration-dependently. Although substantial amounts of koetjapic (**1**) and betulinic (**3**) acids in the crude methanol extracts and fractions of *D. serrata* may affect LPS-induced PGE_2_ production, the presence of other compounds in the extracts and fractions may also contribute to the observed activity. Further studies need to be carried out to investigate the effect of these compounds on COX-2 especially *in vitro* prostaglandin biosynthesis catalysed by COX-2 in cell free assays and expression of COX-2 protein.
